# Optimizing Cattle, Yak, Camel, and Horse Meat Processing: Species‐Sex Physicochemical Drivers

**DOI:** 10.1002/fsn3.71394

**Published:** 2026-01-29

**Authors:** Xueyuan Bai, Yilin Bai, Jing Li, Chaozhi Zhu, Long Xu, Xiaoling Yu, Feng Yin, Ang Ru, Xinghui Wang, Yueyu Bai

**Affiliations:** ^1^ College of Food Science and Technology Henan Agricultural University Zhengzhou China; ^2^ Henan Key Lab of Meat Processing and Quality Safety Control Henan Agricultural University Zhengzhou China; ^3^ Henan Engineering Research Center of for Deep Processing of Beef and Mutton Products Shanghai Haoshou (Henan) Food Co., Ltd. Zhumadian China; ^4^ School of Agricultural Sciences Zhengzhou University Zhengzhou China; ^5^ Institute of Livestock Breeding and Reproduction of Henan University Zhengzhou China

**Keywords:** canonical discriminant analyses, nutrition characteristics, processing characteristics, sex, species

## Abstract

This study assessed species and sex effects on nutritional and processing traits of meat from cattle ( *Bos taurus* ), yak ( *Bos grunniens* ), camel ( *Camelus bactrianus* ), and horse ( *Equus caballus* ). Nutrient characteristics exhibited significant interspecific differences but minimal sex‐related variation. Notably, yak meat exhibited superior nutritional quality—higher protein (20.05%), lower fat (3.13%), richer essential (8.73 mg/g) and flavor (9.23 mg/g) amino acids, as well as elevated eicosapentaenoic acid (EPA, 0.24%), docosahexaenoic acid (DHA, 0.48%), and monounsaturated fatty acids (MUFAs, 52.10%) (*p* < 0.05)—but required tenderization due to high shear force. In contrast, horse meat exhibited greater tenderness and a higher polyunsaturated fatty acid (PUFA) content (9.35%) (*p* < 0.05), though its low water‐holding capacity (WHC) and dark color present processing challenges. Unlike species effect dominating the nutritional and processing traits, sex mainly influenced processing characteristics, as evidenced by the more tender, richer marbled, and brighter meat from females (*p* < 0.05). Overall, nutritional profiles were primarily determined by species, with cattle and horse being similar and distinct from yak and camel. For processing, sex significantly influenced processing traits in yak, camel, and horse, but not in cattle. These findings support the development of tailored processing strategies to better utilize different red meat resources.

## Graphical abstract

Species primarily determines the nutritional and processing traits of cattle, yak, camel, and horse meats; yak stands out for superior nutrition (high protein/EAA/MUFAs) but requires tenderization, while horse offers greater tenderness but has a darker color and low water‐holding capacity. Sex mainly affects processing: female meat is more tender, marbled, and lighter, with significant sex‐specific processing differences in yak, camel, and horse, but not in cattle. Consequently, optimized processing strategies must integrate both species and sex.

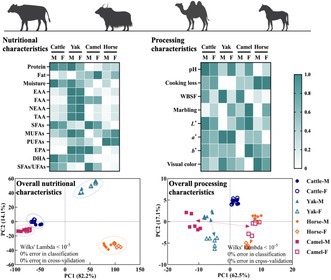

## 1 Introduction

Meat constitutes a globally consumed nutrient‐dense food source, providing essential proteins, lipids, vitamins, and minerals in bioavailable forms (Hocquette 2023; Wood 2023). Numerous studies have focused on enhancing the processing characteristics of the four main meat species—poultry, swine, bovine, and ovine (Savell 2023; Wood 2023). However, due to growing environmental and health concerns related to bovine meat consumption, there is an urgent need to explore alternative red meat species. Notably, yak ( *Bos grunniens* ), horse ( *Equus ferus caballus* ), and camel ( *Camelus bactrianus* ) meats demonstrate significant nutritional potential as sustainable protein sources and show accelerating demand in specialized meat markets (Baba et al. 2021; Li et al. 2023; Stanisławczyk et al. 2021). The optimization of processing protocols for these alternative meats requires understanding not only species‐specific traits but also sex‐related variations—a critical factor for industrial applications (Fresán et al. 2019; Hopkins and Ertbjerg 2023). In commercial practice, sex‐based differences directly influence processing parameters, product segmentation, and market positioning. For instance, female animals often yield more tender and marbled meat, enabling premium product development, whereas meat from males may require specific tenderization strategies (Razmaitė et al. 2021).

In China's western regions, yak, horse, and camel populations have traditionally served as beasts of burden and meat sources. Contemporary agricultural practices increasingly recognize their value in addressing protein security challenges. Tibetan Plateau communities preferentially utilize yak meat for its distinct organoleptic properties, reduced lipid content (Bai et al. 2023), and elevated concentrations of essential fatty acids (Bai et al. 2024; Li et al. 2023). European consumers value horse meat for its favorable amino‐acid score, abundant B‐vitamins and haem iron, and high omega‐3 but low cholesterol content (Beldarrain et al. 2022; Della Malva et al. 2022; Stanisławczyk et al. 2021). Similarly, camel meat presents as a lean protein alternative with demonstrated therapeutic potential (Baba et al. 2021; Si et al. 2022; Zhao et al. 2020).

Processing guidelines are scarce because species‐ and sex‐driven quality differences remain poorly described. Available evidence indicates that yak and horse meats exhibit greater shear force values compared to bovine controls (Stanisławczyk et al. 2021; Wang et al. 2021), while female carcasses demonstrate enhanced intramuscular adipogenesis correlated with improved tenderness metrics (Hopkins and Ertbjerg 2023; Razmaitė et al. 2021). However, the effects of species, sex, and their interaction on meat quality remain poorly understood, yet they are directly relevant to key industrial processes like postmortem aging, raw material segmentation, and functional property prediction. Addressing this gap is therefore critical, as it would enable processors to develop tailored protocols, optimize resource utilization, and ultimately create products targeted to specific market demands. This study therefore aimed to elucidate the effects of species and sex on chemical composition and processing characteristics, with the goal of informing strategies for the optimized utilization and market‐driven development of these alternative red meat resources.

## 2 Materials and Methods

Animal procedures followed the European Directive 2010/63/EU and were approved by the Henan Agricultural University IACUC (20240216).

### 2.1 Meat Samples Collection

Red meat samples were procured from Qinghai Xiahua Halal Meat Products Co. Ltd., with nine biologically independent animals per species‐sex group randomly selected for this study. The detailed traceability information for all animals is provided in Table 1. Upon completion of the standardized Halal slaughter and 24 h postmortem aging (0°C–4°C) protocol across species and sexes, a 3–5 kg sample of the *longissimus thoracis* (LT) muscle was excised solely from the right‐half of each carcass. Subsequently, each LT muscle was sectioned perpendicular to the orientation of the muscle fibers into multiple 5‐cm‐thick steaks, which were randomly allocated to serve as experimental replicates for the various analyses conducted in this study. All steaks were individually vacuum‐packed, frozen at −40°C for 48 h, and then transported to the laboratory under frozen conditions.

**TABLE 1:** Basic information of animals.

Meat sources	Cattle	Yak	Camel	Horse
Breed	Nanyang yellow cattle	Datong yak	Qaidam bactrian camel	Qaidam horse
Latin name	*Bos taurus domesticus*	*Bos grunniens*	*Camelus bactrianus*	*Equus ferus caballus*
Rearing method^a^,, ^b^	Grass‐fed+ concentrated finishing	Grass‐fed+ concentrated finishing	Grass‐fed+ concentrated finishing	Grass‐fed+ concentrated finishing

	Captivity	Pastoral care	Pastoral care	Pastoral care
Slaughter age (year)^c^	2.65 ± 0.27	5.63 ± 0.45	5.56 ± 0.50	5.27 ± 0.39
**Live weight (kg)**				
Male	620.37 ± 40.21	501.26 ± 38.76	670.55 ± 54.16	386.98 ± 29.27
Female	525.66 ± 35.14	389.07 ± 42.32	578.33 ± 46.02	347.65 ± 33.86

^a^ Based on the predominant commercial farming methods for each animal species.

^b^ Intensive fattening for 120 days was conducted with a feedlot finishing diet consisting of (DM basis) 18.0% corn silage, 76.0% shell corn, 5.6% soybean meal, 0.14% limestone, and 0.23% trace mineralized salt.

^c^ According to the average age of commercial slaughter of each animal species.

### 2.2 Chemical Analyses

Semi‐thawed meat samples (thawing at 4°C for 12 h) were processed by removing external fat from the surface and connective tissue. The samples were then minced (3 g) using a specific mincer (S2‐A808, Joyoung Ltd., Shandong, China) to determine moisture content via the drying method at 105°C (Smart Turbo, CEM Corp., USA). Protein was determined by Kjeldahl analysis (Kjeltec 8100, Foss, Denmark) using a 6.25 conversion factor after 24 h thawing at 4°C. The crude fat content was tested using the Soxhlet extraction method with a solvent extraction system (SOX606, Hanon Advanced Technology Group Co. Ltd., Jinan, China).

Amino acid profiles were determined using frozen samples, following the methods described by Tian et al. (2021) with some modifications. One gram of meat was hydrolyzed in 10 mL of 6 M HCl at 105°C under vacuum for 24 h to break down the proteins into free amino acids. After hydrolysis, the mixtures were transferred and diluted to a final volume of 50 mL using 0.02 M HCl. The diluted solutions were mixed, and 1 mL was then transferred to a 6‐cm glass dish and evaporated to dryness in a water bath at 65°C. The resulting residue was dissolved in 2 mL of 0.02 M HCl and then filtered using a 0.22 μm filter. The derivatives were analyzed using a high‐performance liquid chromatography (HPLC) system (LC‐20A, Shimadzu) equipped with a fluorescence detector (RF20A, Shimadzu, Japan) and an AJS‐01 amino acid analytical column (C18, 3 μm, 4.6 × 150 mm; Welch Technology Co. Ltd., Shanghai, China). Individual amino acids were identified by comparing their retention times and fluorescence signals to those of standard amino acids (013–08391, Wako, Tokyo, Japan). The results are expressed as mg/g.

Fatty acid profiles were analyzed as methyl esters (FAMEs) using a method adapted from Bai et al. (2022). Frozen muscle samples (15 g) were ground using a Philips HR2657 grinder (Eindhoven, Netherlands). The ground samples were then mixed with 10 mg of an internal standard (triundecanoin, C11:0), 100 mg of pyrogallic acid, a few boiling chips, 2 mL of 95% ethanol, and 10 mL of 8.3 M HCl. This mixture was hydrolyzed for 40 min in a water bath at 70°C–80°C. After cooling, an additional 10 mL of 95% ethanol was added, and the samples were thoroughly mixed. Triacylglycerols and phospholipids were extracted using a solvent system of diethyl ether and petroleum ether (2:1, v/v) for 1 h. The organic phase was collected and dried under a gentle nitrogen stream. The dried residue was re‐dissolved in 5 mL of a 1:1 chloroform‐diethyl ether mixture and dried again at 40°C under a gentle nitrogen flow. The extracted fat was then esterified by adding 2 mL of 7% boron trifluoride in methanol and toluene, and heating at 100°C for 45 min. Upon cooling to room temperature, the FAMEs were extracted using a system of 5 mL water, 1 mL toluene, and 1 g Na_2_SO_4_. This mixture was vortexed for 1 min and allowed to stand for 5 min. The upper organic layer was collected and then filtered. The FAMEs were analyzed using gas chromatography–mass spectrometry (GC–MS, Agilent 6890A‐5975C, USA) equipped with a CP‐Sil 88 capillary column (100 m × 0.25 mm × 0.25 μm, Chrompack, Middelburg, Netherlands). Fatty acid compositions were determined based on the peak areas of the internal standards and identified using individual and mixed FAME standards (Supelco 37‐component FAMEs mix, Sigma‐Aldrich, Munich, Germany). The results are expressed as percentages of the total fatty acids.

### 2.3 Meat Quality Evaluation

The pH values were measured using a portable pH meter (Testo 205, Lenzkirch, Germany). The electrode was inserted into the steak at a depth of 3 cm, and the measurement was repeated five times. The pH meter was pre‐calibrated using standard buffer solutions with pH values of 4.0 and 7.0. Cooking losses were evaluated using a method based on Bai et al. (2023) with slight modifications. After thawing, the beef samples were trimmed into cuboid blocks measuring 3 cm × 3 cm × 4 cm. The Warner‐Bratzler shear force (WBSF) was determined using a method outlined by Zhao et al. (2022). A single batch of samples (approximately 100 g each) was cooked in a constant‐temperature water bath (Model HH‐8, Nanbei Instrument, China) at 75°C. The samples were placed in foil bags and submerged 5 cm underwater using a metal frame, ensuring they were more than 5 cm away from the walls of the water bath. Cooking continued until the core temperature of the beef reached 70°C. After removing the samples from the water bath, they were cooled in the bags at room temperature for 1 h. Each sample was then cut into 3 cm (parallel to the muscle fibers) × 1 cm × 1 cm cubes, and the maximum shear force of each cube was recorded using a Warner‐Bratzler Meat Shear (Model 235 6X, SALTER Brecknell, GR Manufacturing Corp., USA) at a speed of 10 mm/min. Seven replicates were performed for each sample. The marbling scores and meat color were evaluated by a trained panel of seven university faculty and staff. The marbling scores were rated from 1 to 10, where 1 = devoid (PD), 2 = traces (TR), 3 = slight (SL), 4 = small (SM), 5 = modest (MT), 6 = moderate (MD), 7 = slightly abundant (SLAB), 8 = moderately abundant (MAB), 9 = abundant, and 10 = very abundant (VAB). The meat color was rated from 1 to 5, where 1 = slightly light red, 2 = moderately light red, 3 = light red, 4 = slightly dark red, and 5 = dark red (Norman et al. 2003). The instrumental color measurements of the thawed samples were conducted with slight modifications to the method described by Zhao et al. (2022). The CIE L* (brightness), a* (redness), and b* (yellowness) values were assessed using a portable colorimeter (YS3010, Threenh Technology Corp., China) on the freshly cut surfaces of the meat. The meat was allowed to stand at 4°C for 30 min to obtain myoglobin oxygenation before measurement, with the D65 illuminant, an 8 mm aperture, and a 10° observer angle. The colorimeter was pre‐calibrated. Five random points on each sample, avoiding marbling and connective tissues, were selected for determination.

### 2.4 Statistical Analysis

The linear mixed models (LMM) were used to analyze the physicochemical indicators of red meat and were run on SPSS software (version 26, IBM Corp., USA). Fixed effects for full models included animals (cattle/yak/horse/camel), sex (male/female), and their interactions (denoted by animals × sex). The full model of each index also included random terms (rearing method, farming area, slaughter age, and detection order) and covariates. Live weight was used as a covariate in the initial models of protein, moisture, and fat content. Protein and fat content were used as covariates in the initial models of amino acid and fatty acid indices, respectively. Live weight, protein, moisture, and fat content were included as covariates in the initial models of processing indices. Nonsignificant fixed terms (*p* > 0.05) were excluded from each model. The fitted models were used to predict the mean. Results were expressed as mean ± standard errors (*n* = 9) with *p* < 0.05 considered significantly different. Critically, non‐significant effects (*p* > 0.05 for gender and interaction term) were observed in LMMs of amino acid profile and fatty acid indices, eliminating these terms and confirming species as the singular significant predictor (*p* < 0.05) that remained in the final LMMs.

Parameters were subjected to canonical discriminant analysis (CDA) to provide an overview of the nutritional and processing characteristics of red meat from different origins. Before CDA, the data were pre‐processed using min‐max normalization in Excel 2019 (Microsoft Corporation, USA). CDA was then applied separately to two sets of nutritional and processing indices to discriminate between meat species and sex. The nutrition set initially included all parameters of meat proximate composition, amino acid, and fatty acid profiles. Sixteen parameters (protein, moisture, fat, Met, Val, Lys, Ile, Phe, Leu, Thr, His, Asp, Ser, Glu, Pro, and Gly) were retained in the final CDA model. All eight parameters (pH, cooking loss, shear force, marbling score, L*, a*, b* and visual color) of processing characteristics were also retained in the final CDA model. The results were visualized using Prism software (Version 10.4, GraphPad Software, San Diego, CA, USA).

## 3 Results and Discussion

### 3.1 Chemical Composition

**FIGURE 1:**
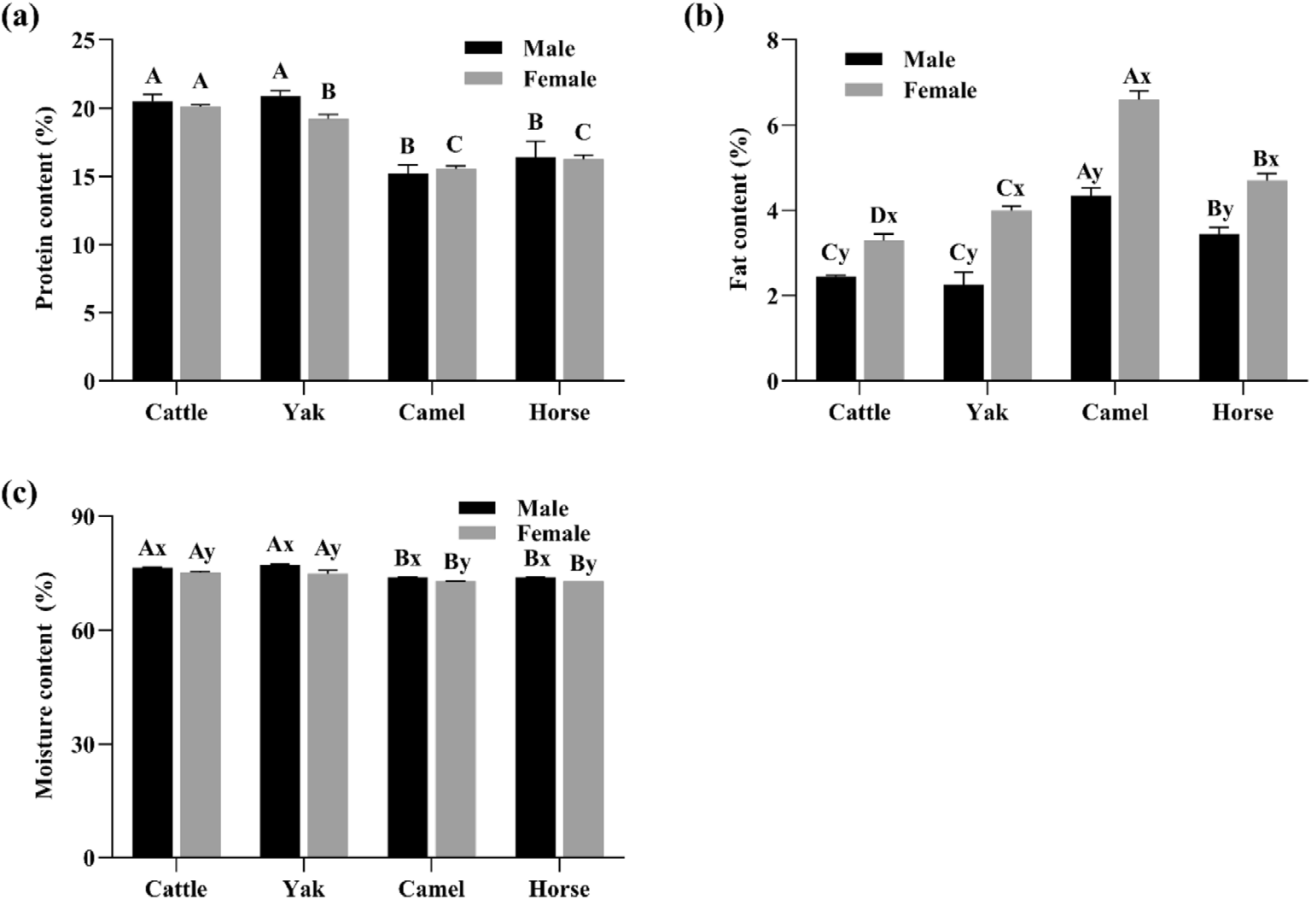
Effects of species and sex on the (a) protein (%), (b) fat (%), and (c) moisture content (%) in *longissimus thoracis* (LT) muscle (*n* = 9). The data represent the effects of species and sex on individual traits, and the results were expressed as mean ± standard errors. Uppercase letters (A–D) indicate significant differences (*p* < 0.05) among species within the same sex, while lowercase letters (x, y) denote significant differences between sexes within the same species. This annotation convention is maintained throughout subsequent figures.

The protein, fat, and moisture within the meat matrix altered across animal species and sexes (*p* < 0.05, Figure 1) except for the protein content between sexes (*p* > 0.05, Figure 1a). No interaction terms were detected in the LMM analyses (*p* > 0.05, Table S1). Specifically, compared with cattle and yak, horse and camel LT muscles, regardless of sex, exhibited lower protein and moisture content but higher fat content (*p* < 0.05, Figure 1). Notably, significant sex differences existed in water content and fat deposition (*p* < 0.05). The protein, fat, and moisture content of the four meat types (cattle, yak, horse, and camel) generally align with previously reported ranges for these species (Baba et al. 2021; Lorenzo et al. 2014; Zhang et al. 2016). Female animals across species tend to deposit more fat, consistent with previous findings that estrogen influences lipid accumulation (Park et al. 2018). However, some results deviate from prior studies. For example, grain‐fed cattle meat did not have higher fat content than grass‐fed yak and camel meat, contrary to expectations (Li et al. 2023; Zhang et al. 2016). This difference might be attributable to the compensatory effect of slaughter age on fat deposition. Older yaks, despite grazing naturally, accumulate fat similarly to grain‐fed cattle because of their longer growth periods. This age‐related lipid storage likely offsets the typically leaner profile of pasture‐raised systems. Contrary to the results of the present study, Tateo et al. (2008) found male horses have higher protein and lipid percentages and Abdelhadi et al. (2017) reported male‐biased protein content. This discrepancy may be attributable to differences in the studied breeds and slaughter ages.

### 3.2 Amino Acid Composition

The amino‐acid profiles of cattle, yak, camel, and horse LT muscles revealed distinct nutritional and technological signatures. Meat species significantly influenced amino acid composition (*p* < 0.05, Table 2), while sex and its interaction with species did not (*p* > 0.05, Table S2). Specifically, yak meat stood out with greater content of total amino acids (TAA), essential amino acids (EAA), including Met, Val, Lys, Ile, Leu, Thr, and His, and non‐essential amino acids (NEAA), namely, Asp, Ser, Glu, Pro, Tyr, and Arg (*p* < 0.05). In addition, it contained more abundance of His, Arg, and Ala and branched‐chain amino acids (Leu, Val, and Ile) (*p* < 0.05), making it particularly advantageous for children who required collagen synthesis and athletes or convalescent individuals who required rapid muscle‐protein synthesis (Bassit et al. 2000; Li et al. 2023). This aligns with previous findings by Zhang (2009) who emphasized that yak meat contains elevated Asp and Glu—key umami‐enhancing amino acids—which directly contribute to its superior sensory qualities over conventional beef.

**TABLE 2:** Amino acid profile (mg/g) of *longissimus thoracis* muscle of cattle, yak, horse, and camel (*n* = 18).

Indices	Cattle	Yak	Camel	Horse	*p*
Met	0.52 ± 0.01^b^	0.65 ± 0.02^a^	0.51 ± 0.02^b^	0.51 ± 0.01^b^	***
Val	0.92 ± 0.01^b^	1.13 ± 0.02^a^	0.90 ± 0.02^b^	0.90 ± 0.02^b^	***
Lys	1.86 ± 0.04^b^	2.10 ± 0.03^a^	1.81 ± 0.06^b^	1.81 ± 0.04^b^	***
Ile	0.90 ± 0.02^b^	1.04 ± 0.02^a^	0.87 ± 0.03^b^	0.88 ± 0.03^b^	***
Phe	0.90 ± 0.01	0.92 ± 0.02	0.93 ± 0.03	0.91 ± 0.03	NS
Leu	1.58 ± 0.03^b^	1.88 ± 0.03^a^	1.54 ± 0.05^b^	1.58 ± 0.04^b^	***
Thr	0.85 ± 0.02^b^	1.01 ± 0.03^a^	0.84 ± 0.03^b^	0.84 ± 0.02^b^	***
His	0.70 ± 0.02^c^	0.98 ± 0.02^a^	0.71 ± 0.03^c^	0.84 ± 0.07^b^	***
Asp	0.69 ± 0.01^c^	2.04 ± 0.04^a^	1.73 ± 0.06^b^	0.70 ± 0.02^c^	***
Ser	3.22 ± 0.07^a^	0.88 ± 0.03^c^	0.68 ± 0.03^d^	3.01 ± 0.05^b^	***
Glu	0.70 ± 0.02^c^	3.34 ± 0.04^a^	3.13 ± 0.10^b^	0.68 ± 0.01^c^	***
Pro	0.81 ± 0.01^a^	0.85 ± 0.02^a^	0.71 ± 0.03^b^	0.80 ± 0.02^a^	**
Gly	1.11 ± 0.02^a^	0.90 ± 0.02^b^	0.76 ± 0.03^c^	1.08 ± 0.02^a^	***
Ala	1.77 ± 0.03^a^	1.30 ± 0.01^b^	1.07 ± 0.04^c^	1.72 ± 0.04^a^	***
Tyr	0.69 ± 0.02^bc^	0.84 ± 0.01^a^	0.66 ± 0.02^c^	0.74 ± 0.02^b^	***
Arg	1.33 ± 0.03^b^	1.46 ± 0.02^a^	1.30 ± 0.05^b^	1.27 ± 0.03^b^	**
TAA	18.53 ± 0.32^b^	21.50 ± 0.07^a^	18.14 ± 0.60^b^	18.28 ± 0.45^b^	***
EAA	7.53 ± 0.13^b^	8.73 ± 0.05^a^	7.39 ± 0.24^b^	7.44 ± 0.20^b^	***
NEAA	11.00 ± 0.19^b^	12.77 ± 0.09^a^	10.76 ± 0.36^b^	10.84 ± 0.26^b^	***
FAA	5.58 ± 0.11^c^	9.23 ± 0.09^a^	7.99 ± 0.27^b^	5.45 ± 0.11^c^	***
EAA/TAA (%)	40.66 ± 0.06	40.61 ± 0.29	40.72 ± 0.05	40.69 ± 0.08	NS
EAA/NEAA (%)	68.52 ± 0.16	68.41 ± 0.84	68.70 ± 0.16	68.62 ± 0.22	NS
FAA/TAA (%)	30.13 ± 0.16^c^	42.93 ± 0.30^b^	44.03 ± 0.08^a^	29.86 ± 0.14^c^	***

*Note:* The values of the above indicators were generated as individual traits in terms of animal species as the effects of gender and its interaction with animal species on amino acid composition having a significant level of *p* > 0.05. a–d: indicates the differences in the same row with significance at *p* < 0.05.

Abbreviations: EAA, essential amino acids; FAA, flavor amino acids, including Asp, Glu, Gly, Ala and Arg; NEAA, non‐essential amino acids; NS, non‐significant; TAA, total amino acids.

** *p* < 0.01.

*** *p* < 0.001.

In contrast, cattle, camel, and horse clustered at lower EAA and TAA values (*p* < 0.05), but differed markedly in their flavor‐forming free amino acids (FAAs) (*p* < 0.05). Camel muscle showed the higher proportion of FAA/TAA, driven by high Glu and Asp (*p* < 0.05), yet its EAA level was modest, suggesting that fermentation or drying processes (e.g., air‐dried camel sausage) would both intensify umami and improve protein digestibility (Chen et al. 2024). These attributes align with the opinion of Lorenzo et al. (2014), who highlighted camel meat's suitability as a “dietetic” option, particularly in regions seeking nutritious red meats. Meanwhile, contrary to previous findings reporting the enrichment of EAA (Lys, Leu, Arg) in camel meat and (Leu and Lys) in horse meat, our study revealed no statistically significant superiority in these specific EAA profiles compared to other red meat types investigated (Abdelhadi et al. 2017; Della Malva et al. 2022).

### 3.3 Fatty Acid Composition

Similarly, the LT muscles of cattle, yak, horse and camel exhibited inter‐species differences in fatty‐acid composition (*p* < 0.05, Table 3), which was not affected by sex or the interaction term (*p* > 0.05, Table S3). Compared to cattle and camel meats, horse and yak meats contain higher levels of MUFAs and PUFAs and lower levels of SFAs and SFAs/UFAs (*p* < 0.001), offering superior nutritional benefits. Notably, yak presented the higher eicosapentaenoic (EPA, C20:5n3) and docosahexaenoic (DHA, C22:6n3) content, demonstrating anti‐inflammatory, neuroprotective, and cardiometabolic regulatory properties as evidenced by in vivo studies (Li et al. 2023); whereas horse displayed an unusually elevated γ‐linolenic acid (C18:3n6) rarely reported in muscle foods.

**TABLE 3:** Fatty acid profile (% of total FA) of *longissimus thoracis* muscle of cattle, yak, horse, and camel (*n* = 18).

Indices	Cattle	Yak	Camel	Horse	*p*
C14:0	7.59 ± 0.25^a^	2.07 ± 0.06^d^	3.07 ± 0.13^c^	4.48 ± 0.58^b^	***
C16:0	28.21 ± 0.96^a^	14.55 ± 0.23^c^	26.00 ± 0.27^b^	29.50 ± 0.44^a^	***
C18:0	14.81 ± 1.27^c^	21.57 ± 0.19^b^	27.53 ± 0.80^a^	3.50 ± 0.11^d^	***
**SFAs**	51.67 ± 0.72^a^	39.00 ± 0.29^b^	53.11 ± 3.38^a^	36.43 ± 0.82^b^	***
C14:1	0.13 ± 0.02^b^	0.33 ± 0.04^a^	0.17 ± 0.01^b^	0.30 ± 0.02^a^	***
C16:1	3.03 ± 0.27^c^	5.04 ± 0.11^b^	2.79 ± 0.16^c^	6.83 ± 0.15^a^	***
C17:1	0.30 ± 0.01^b^	0.67 ± 0.02^a^	0.33 ± 0.02^b^	0.35 ± 0.03^b^	***
C18:1n9c	37.70 ± 0.95^b^	45.39 ± 0.47^a^	32.84 ± 1.40^c^	35.63 ± 0.68^b^	***
C20:1n9	0.26 ± 0.04^a^	0.34 ± 0.02^a^	0.13 ± 0.01^b^	0.16 ± 0.02^b^	***
C22:1n9	0.64 ± 0.42^c^	0.44 ± 0.11^c^	4.59 ± 0.02^b^	6.64 ± 0.46^a^	***
C24:1n9	1.91 ± 1.09^b^	0.15 ± 0.02^c^	3.21 ± 0.45^a^	3.67 ± 0.26^a^	***
**MUFAs**	42.50 ± 1.07^b^	52.10 ± 0.46^a^	37.44 ± 1.51^c^	48.86 ± 1.30^a^	***
C18:2n6c	1.71 ± 0.15^b^	3.01 ± 0.05^a^	1.94 ± 0.32^b^	3.01 ± 0.15^a^	***
C18:3n6	0.48 ± 0.07^b^	0.45 ± 0.06^b^	0.75 ± 0.08^b^	7.04 ± 0.56^a^	***
C20:2	0.02 ± 0.01^b^	0.11 ± 0.03^a^	ND	0.05 ± 0.01^b^	*
C20:3	0.34 ± 0.11^ab^	0.36 ± 0.04^a^	0.10 ± 0.02^c^	0.18 ± 0.01^bc^	**
C20:4n6	0.08 ± 0.02^c^	1.20 ± 0.04^a^	0.32 ± 0.11^b^	0.17 ± 0.03^bc^	***
C20:5n3	ND	0.24 ± 0.04^a^	0.18 ± 0.05^a^	0.05 ± 0.01^b^	**
C22:6n3	0.50 ± 0.05^a^	0.48 ± 0.01^a^	0.05 ± 0.01^b^	0.04 ± 0.00^b^	***
**PUFAs**	3.11 ± 0.19^c^	5.85 ± 0.23^b^	3.35 ± 0.57^c^	9.35 ± 0.94^a^	***
SFAs/UFAs	1.14 ± 0.03^b^	0.67 ± 0.01^c^	1.33 ± 0.11^a^	0.63 ± 0.01^c^	***
PUFAs/SFAs	0.06 ± 0.01^c^	0.15 ± 0.01^b^	0.06 ± 0.01^c^	0.25 ± 0.02^a^	***

*Note:* The values of the above indicators were generated as individual traits in terms of animal species as the effects of gender and its interaction with animal species on the composition of fatty acids having a significant level of *P* > 0.05. a–d: indicates the differences in the same row with significance at *p* < 0.05.

Abbreviations: MUFAs, total monounsaturated fatty acids; ND, not detected; NS, non‐significant; PUFAs, total polyunsaturated fatty acids; SFAs, total saturated fatty acids; UFA, unsaturated fatty acids.

* *p* < 0.05.

** *p* < 0.01.

*** *p* < 0.001.

These findings integrate and extend several established paradigms. First, they corroborate Zhang (2009), who demonstrated that pasture‐fed herbivores accumulate plant‐derived fatty acids via ecological niche specialization, and Wood et al. (2008), who showed that prolonged natural grazing augments the deposition of beneficial long‐chain n‐3 fatty acids, including DHA and EPA. In the present study, yak exemplifies this principle: its adipose tissue contains the highest combined DHA and EPA within a ruminant matrix while simultaneously delivering elevated monounsaturates, rendering it the most nutritionally balanced of the four species. Conversely, the high SFA content observed in cattle aligns with previous reports that concentrate‐based diets elevate SFAs (Penner et al. 2011; Cobellis et al. 2016), underscoring the feed‐driven divergence in muscle lipid composition. Across all four meats, however, PUFA/SFA ratios remained below the WHO‐recommended range of 0.4–1.0 (Gillis et al. 2004; Rawdah et al. 1994; Woods and Fearon 2009), potentially limiting their cardioprotective efficacy. Horse meat was the notable exception, exhibiting the higher PUFA content and a unique dominance of C18:3n6—a γ‐linolenic acid seldom abundant in conventional red meats. This peculiarity likely reflects evolutionary adaptations to arid‐zone foraging on drought‐resistant flora rich in ω‐6 precursors (Lorenzo et al. 2014). Camel and cattle, characterized by energy‐dense, SFA‐rich profiles, remain suitable for high‐calorie applications but are less favorable for consumers with dyslipidaemic risk.

Processing implications follow directly from these compositional traits. Yak's elevated unsaturated fatty acids confer excellent cold‐chain stability; thus, vacuum‐aged steaks or sous‐vide preparations better preserve n‐3 PUFAs while maintaining juiciness. Horse's high PUFA and MUFA load necessitates antioxidant strategies (e.g., α‐tocopherol or rosemary extracts) and favors oxidative‐tolerant processes such as smoking or air‐drying (Ribeiro et al. 2019). Cattle and camel, with robust SFA content, withstands high‐temperature grilling and forms desirable flavor compounds via Maillard reactions, making it ideal for burgers and steaks.

### 3.4 Meat Color Characteristics

**FIGURE 2:**
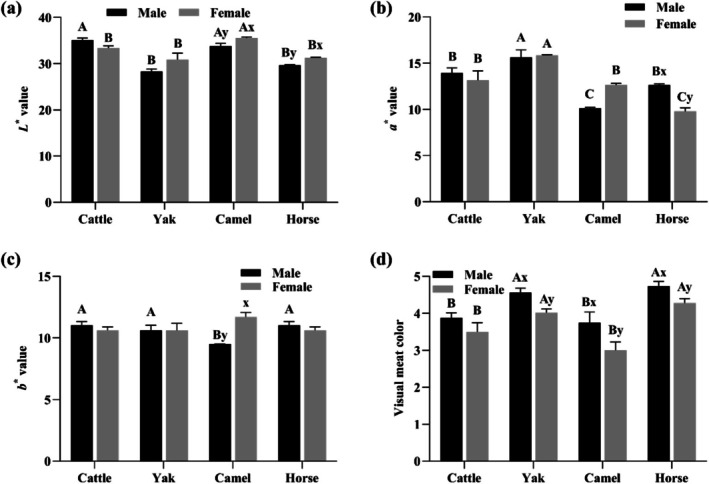
Effects of species and sex on (a) lightness (L*), (b) redness (a*), (c) yellowness (b*), and (d) visual color score (1–5 scale) of LT muscle (*n* = 9).

Comprehensive colorimetric evaluation of the LT muscles across cattle, yak, camel and horse revealed significant inter‐specific divergence and gender effects (*p* < 0.05, Figure 2). Camel meat presented brighter (L*) surface than yak and horse regardless of sex (*p* < 0.05, Figure 2a). Female horse and camel meats exhibited significantly higher lightness values compared to their male counterparts (*p* < 0.05, Figure 2a), which may relate to the fat deposition and marbling score. Yak meat consistently exhibited higher redness (a*) values across sexes (*p* < 0.05, Figure 2b), attributed to elevated myoglobin concentrations in high‐altitude‐adapted muscles (Bai et al. 2023). Interestingly, male horse meat exhibited a redder surface, possibly due to higher physical activity levels compared to females (Faustman et al. 2023). Camel meat has lower yellowness (b*) within the male group (*p* < 0.05, Figure 2c); however, female meat shared similar yellowness across species (*p* > 0.05). The visual color of meat was significantly influenced by species, sex, and their interaction (*p* < 0.05, Figure 2d; Table S4), with overall scores ranging from light red to slight dark red. Yak and horse meats (slight dark red) exhibited higher visual color scores than cattle and camel meats (light red) regardless of sex, while female ones demonstrated lower scores (more toward consumer‐preferred light red) compared to their male counterparts (*p* < 0.05, Figure 2d) except for cattle. These color variations impact consumer acceptance and necessitate species‐specific packaging or antioxidant treatments (e.g., rosemary extract for camel meat) to mitigate oxidation (Baba et al. 2021; Bai et al. 2022).

### 3.5 Meat pH and Cooking Loss

**FIGURE 3:**
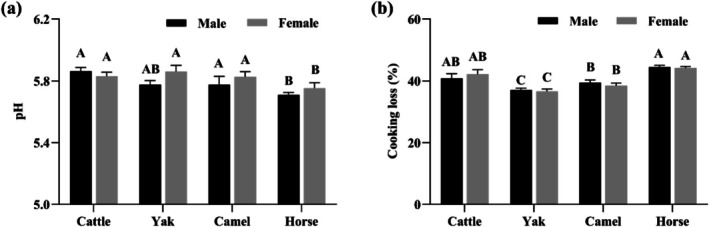
Effects of species and sex on (a) pH and (b) cooking loss (%) of LT muscle (*n* = 9).

The pH of meat is a critical parameter affecting protein charge states and enzymatic activity, which in turn influence water‐holding capacity (WHC), tenderness, and color (Hopkins and Ertbjerg 2023). It was not sex (*p* > 0.05) but species that significantly influenced meat pH and cooking loss (*p* < 0.05, Figure 3). Horse meat displayed lower pH values compared to cattle and camel (*p* < 0.05, Figure 3a), while it exhibited greater WHC than yak and camel (*p* < 0.05, Figure 3b) as expected. The observed pH and WHC variations were consistent with previously reported ranges (Baba et al. 2021; Lorenzo et al. 2014; Zhang et al. 2016). High ultimate pH maintains myofibrillar spacing via electrostatic repulsion, whereas low pH induces shrinkage and water loss due to diminished charge repulsion near the isoelectric point (pI 5.0 ~ 5.2) (Warner 2023). These variations of pH may stem from species‐specific muscle glycogen reserve and postmortem glycolysis rates (Abdelhadi et al. 2017). In contrast, sex differences within species were inconsistent: camel and yak ultimate pH showed no sex variation (Abdelhadi et al. 2017; Zhang 2009), whereas stallion meat had higher pH than mares (Razmaitė et al. 2021). Additionally, previous reports have indicated that beef WHC is not influenced by sex (Razmaitė et al. 2021).

### 3.6 Marbling Scores and Meat Tenderness

**FIGURE 4:**
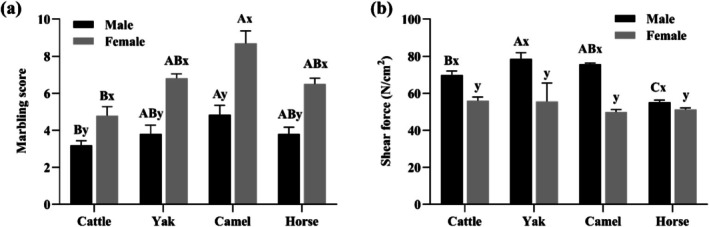
Effects of species and sex on (a) marbling score (1–10 scale) and (b) shear force (N/cm^2^) of LT muscle (*n* = 9).

Marbling scores and tenderness are key indices in meat evaluation that respectively measure the meat's intramuscular fat distribution and hardness, which jointly influence the eating quality and consumer acceptance of meat products (Hopkins and Ertbjerg 2023). Marbling scores and tenderness were affected by both species and sex (*p* < 0.05, Figure 4). Female meat universally exhibited higher marbling scores and tenderness than males (*p* < 0.05, Figure 4), likely attributable to estrogen‐driven fat deposition (Xiong et al. 2021). In Figure 4a, camel meat showed higher marbling scores than beef (*p* < 0.05), and interestingly, yak and horse meat had intermediate scores. This contradicts the traditional view that horse meat is very lean (Lorenzo et al. 2010, 2014). In male animals, marbling scores varied from TR to SM. Camel meat only reached the TR level, yak and beef attained the SL grade, and horse meat achieved the SM grade. Female animals, however, generally had higher marbling scores, ranging from SM in cattle to MAB in camel.

Tenderness variations are closely related to animal gender. In male animals, yak meat was harder while horse meat was tenderer (*p* < 0.05, Figure 4b); however, in female animals, there's no difference in tenderness across species (*p* > 0.05, Figure 4b). Notably, female animal meat is more tender than that of males (*p* < 0.05). Generally, yak, camel, and horse were slaughtered at older ages than cattle, supposed to possess harder meat, as increasing age raises total collagen content and/or cross–linking, toughening the meat (Abdelhadi et al. 2017; Bai et al. 2022; Hopkins and Ertbjerg 2023). Tenderness of male yak and camel meat met this expectation. However, horse meat exhibited higher tenderness than cattle, a characteristic likely due to its high intramuscular fat content mirrored by marbling scores, which offset age‐related toughening (Bai et al. 2022). This phenomenon also resulted in a non‐significant difference in tenderness among female species.

The physicochemical properties of meat from cattle, yak, camel, and horse highlighted distinct processing suitability for each species. Cattle meat, characterized by high protein content, low fat content, and elevated SFAs is ideal for traditional cured products or jerky, where protein integrity and flavor development are prioritized. Yak meat, distinguished by the higher FAA proportion and intermediate fat content, is particularly suitable for fermented or smoked products (e.g., sausages), leveraging its umami‐enhancing amino acids to amplify sensory appeal. However, its high shear force necessitates tenderization techniques, such as enzymatic marination, prior to thermal processing. Camel meat, with the higher crude fat and moisture content, is optimal for emulsified products (e.g., hot dogs), where fat acts as a binding agent and moisture retention is critical. Its elevated PUFA/SFA ratio and marbling scores further enhance juiciness in grilled or roasted applications. Conversely, horse meat, despite lower protein content, exhibits the higher PUFAs and the lower shear force, making it suitable for minimally processed products (e.g., sous‐vide cuts or cold cuts) that preserve its oxidative‐labile PUFAs. The pronounced sex‐based differences in fat content suggest that female meats are better suited for slow‐cooked dishes, while leaner male meats align with quick‐cooking methods. These species‐specific recommendations should integrate antioxidant strategies (e.g., rosemary extract for camel meat) and textural optimization (e.g., mechanical tenderization for yak) to address compositional vulnerabilities while maximizing nutritional and sensory outcomes.

### 3.7 Multivariate Discrimination of Meat Nutritional and Processing Characteristics by CDAs

**FIGURE 5:**
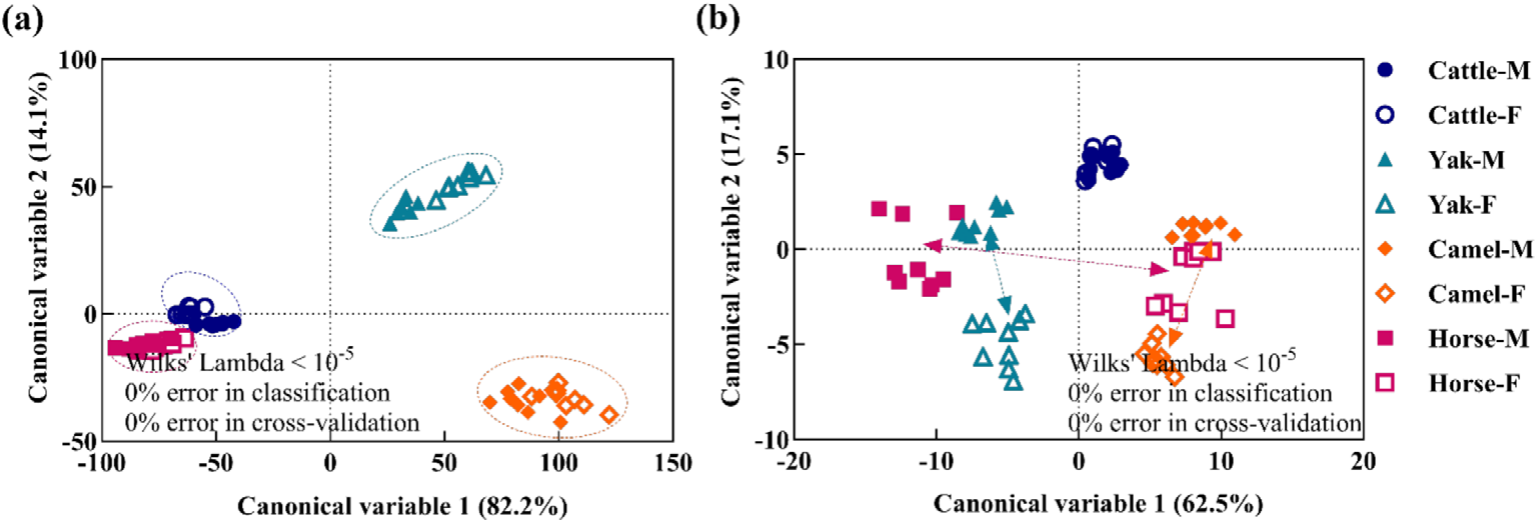
Canonical discriminant analysis (CDA) for discrimination among species and sex using (a) nutritional and (b) processing characteristics of the LT muscle (*n* = 9). Final nutritional CDA model included 16 parameters (protein, moisture, fat, Met, Val, Lys, Ile, Phe, Leu, Thr, His, Asp, Ser, Glu, Pro, and Gly) while the processing model retained eight parameters (pH, cooking loss, shear force, marbling score, L*, a*, b*, and visual color). Labels combine species and sex (e.g., Cattle‐F and Cattle‐M for female and male cattle).

CDA models were constructed based on comprehensive nutritional and processing indices to differentiate meat sources by species and sex (Figure 5). Both models demonstrated exceptional discriminatory power, achieving 100% accuracy in classification and cross‐validation procedures.

The nutritional characteristics model (Figure 5a) revealed two significant canonical dimensions. Canonical variable 1 (CAN1) accounted for 82.2% of inter‐group variance, effectively distinguishing horse and bovine meats from yak and camel products. Canonical variable 2 (CAN2) explained 14.1% of the residual variance, enabling further differentiation between horse and bovine samples. CDA analysis reveals a closer nutritional alignment between cattle and horse meats, while distinct compositional divergences exist relative to yak or camel meats. Notably, while interspecies nutritional profiles exhibited marked divergence, sex‐based variations within species demonstrated minimal statistical separation, suggesting potential for nutritional complementarity between species in dietary applications.

Correspondingly, the processing characteristics model (Figure 5b) demonstrated distinct separation patterns. CAN1 captured 62.5% of variance, primarily roughly discriminating species‐specific processing requirements, together with CAN2 (17.1% variance) revealing significant sex‐mediated heterogeneity across species. Sex significantly influenced horse meat processing traits, with male camels exhibiting yak‐like processing properties, while females demonstrated closer alignment with camel meat characteristics. Contrarily, cattle processing characteristics demonstrated negligible sex differentiation, contrasting with other species.

This multivariate analysis highlights fundamental divergence in quality determinants between bovine and alternative red meats. The pronounced sexual dimorphism observed in yak, camel, and horse species necessitates sex‐specific processing methods, particularly for horse products where sexual dimorphism exceeded interspecies variation. To our knowledge, this represents the first comprehensive comparison of these four species considering both nutritional and technological parameters. These findings provide empirical justification for developing customized processing frameworks that account for both interspecific and sex heterogeneity in alternative meat systems.

Observed deviations in shear force, ultimate pH, and WHC translate directly into controllable processing levers. Yak meat's inherently high hardness necessitates pre‐tenderization—blade penetration or enzyme injection ensures a consumer‐friendly texture without relying on excessive salt or phosphates, supporting clean‐label positioning (Crowley 2023). In contrast, horse meat's naturally low pH accelerates marinade uptake but also decreases WHC; a phosphate‐free dip enriched with plant antioxidants can halve purge and significantly improve color stability under retail lighting (Teixeira et al. 2025; Weiss et al. 2010). Camel meat, with its elevated intramuscular fat, acts as an internal antioxidant reservoir, allowing processors to shorten postmortem aging duration while still meeting lipid oxidation thresholds for sliced, cured products (Wu et al. 2022; Dragoev 2024). Compared with cattle, yak, horse, and camel meats all present higher oxidative potential. High‐altitude yak exhibits elevated myoglobin, whose autoxidation synergistically drives lipid oxidation, accelerating spoilage (Bai et al. 2022). Horse and camel are intrinsically fatter; especially, camel having elevated PUFA, rich in conjugated double bonds, is highly vulnerable to reactive oxygen species (ROS); once depleted, it would transform into a free radical, shortening shelf‐life (Sheard et al. 2000). Consequently, antioxidant interventions during processing, packaging, and storage are essential for these alternative red meats.

Sex‐based segregation offers an additional control point. Female animals typically exhibit higher intramuscular fat, which provides a buffer against overcooking in emulsified products, whereas leaner male meat is better suited for low‐water activity dried meats (Mediani et al. 2022). Integrating these species‐ and sex‐specific parameters into standard operating procedures enhances process control, reduces batch variability, and supports the development of differentiated, ethnically certified product lines that meet growing consumer demand for alternative red meats.

## 4 Conclusion

The present study investigated the nutritional and processing properties of LT muscles across four red meat species: cattle, yak, camel, and horse. The results indicated that species exerted fundamental influence on nutritional and processing aspects, whereas sex primarily affected processing characteristics such as marbling, meat tenderness, and color. Yak meat demonstrated a nutritionally superior profile, characterized by elevated protein content, reduced fat content, and optimal amino acid and FA compositions, especially EPA and DHA, with processing optimization primarily focused on tenderization interventions. Horse meat possessed higher MUFAs, PUFAs, and tenderness, and processing requirements should be focused on its lower WHC and dark red color. Further CDAs analyses suggested, in terms of overall nutritional traits, cattle and horse meats exhibited comparable nutritional profiles, differing significantly from yak and camel, with minimal sex‐based nutritional differences; however, in processing characteristics, substantial sex‐specific variations emerged as female horse meat demonstrated processing behavior analogous to yak, while mare meat aligned with camel meat, contrasting with the consistent processing properties observed across sexes in cattle. In conclusion, meat species significantly affects nutritional and processing traits, but sex differences should be considered during processing. Processors should therefore adopt species‐ and sex‐specific protocols: yak and horse meats of high shear force require mechanical or enzymatic tenderization; well‐marbled cuts from female carcasses are reserved for premium fresh markets; and packaging atmosphere and antioxidant levels must be aligned with the fatty‐acid profile to extend shelf life and maximize profit.

## Author Contributions

**Xueyuan Bai:** writing – original draft, conceptualization, methodology. **Yilin Bai:** funding acquisition, validation, data curation. **Jing Li:** funding acquisition, validation, data curation. **Chaozhi Zhu:** methodology, investigation, validation. **Long Xu:** visualization, writing – original draft. **Xiaoling Yu:** supervision, writing – review and editing. **Feng Yin:** resources. **Ang Ru:** resources. **Xinghui Wang:** formal analysis, software. **Yueyu Bai:** writing – review and editing, project administration.

## Conflicts of Interest

The authors declare no conflicts of interest.

## Supporting information


**Table S1:** Proximate composition of *longissimus thoracis* muscle of female and male cattle, yak, camel, and horse (*n* = 9).
**Table S2:** Amino acid profile (mg/g) of *longissimus thoracis* muscle of female and male cattle, yak, camel, and horse (*n* = 9).
**Table S3:** Fatty acid profile (% of total FA) of *longissimus thoracis* muscle of female and male cattle, yak, camel, and horse (*n* = 9).
**Table S4:** The processing characteristics of *longissimus thoracis* muscle of female and male cattle, yak, camel, and horse (*n* = 9).

## Data Availability

All data included in the present study are available upon request by contact with the corresponding author. The analysis script used in this work is provided in Supporting Information S1.
