# Supplementary material for: Optimizing Cattle, Yak, Camel, and Horse Meat Processing: Species‐Sex Physicochemical Drivers
Source: Food Sci Nutr. 2026 Jan 29;14(2):e71394. doi: 10.1002/fsn3.71394 (PMC12853317; doi:10.1002/fsn3.71394)
Supplement: Supplementary file 1 — Table S1: Proximate composition of longissimus thoracis muscle of female and male cattle, yak, camel, and horse (n = 9). Table S2: Amino acid profile (mg/g) of longissimus thoracis muscle of female and male cattle, yak, camel, and horse (n = 9). Table S3: Fatty acid profile (% of total FA) of longissimus thoracis muscle of female and male cattle, yak, camel, and horse (n = 9). Table S4: The processing characteristics of longissimus thoracis muscle of female and male cattle, yak, camel, and horse (n = 9). [file FSN3-14-e71394-s001.docx]

Table captions

Table S1. Proximate composition of *longissimus thoracis* muscle of female and male cattle, yak, camel and horse (n = 9).

Table S2. Amino acid profile (mg/ g) of *longissimus thoracis* muscle of female and male cattle, yak, camel and horse (n = 9).

Table S3. Fatty acid profile (% of total FA) of *longissimus thoracis* muscle of female and male cattle, yak, camel and horse (n = 9).

Table S4. The processing characteristics of *longissimus thoracis* muscle of female and male cattle, yak, camel and horse (n = 9).

| Table S1. Proximate composition of *longissimus thoracis* muscle of female and male cattle, yak, camel and horse (n = 9). | | | | | | | | | |
| --- | --- | --- | --- | --- | --- | --- | --- | --- | --- |
| Indices | Sex | Species | | | |  | *P*-values | | |
|  |  | Cattle | Yak | Camel | Horse |  | Species | Sex | Species × Sex |
| Protein content | M | 20.51±0.51^A^ | 20.89±0.40^A^ | 17.20±0.64^B^ | 18.40±0.35^B^ |  | *** | NS | NS |
| (%) | F | 20.10±0.14^A^ | 19.22±0.30^B^ | 17.55±0.20^C^ | 18.25±0.32^C^ |  |  |  |  |
| Crude fat content | M | 2.45±0.04^Cy^ | 2.26±0.28^Cy^ | 4.35±0.09^Ay^ | 3.45±0.03^By^ |  | *** | *** | NS |
| (%) | F | 3.29±0.16^Dx^ | 4.00±0.10^Cx^ | 6.60±0.06^Ax^ | 4.70±0.06^Bx^ |  |  |  |  |
| Moisture content | M | 76.57±0.04^Ax^ | 76.20±0.26^Ax^ | 73.98±0.08^Bx^ | 73.98±0.08^Bx^ |  | *** | *** | NS |
| (%) | F | 75.28±0.26^Ay^ | 74.89±1.00^Ay^ | 72.90±0.07^By^ | 72.97±0.01^By^ |  |  |  |  |
| The data of protein, moisture and crude fat content were generated as individual traits in terms of species and sex. A-D Different letters indicate significant differences (*P* < 0.05) among species while x-y between sexes. M, male; F, female; NS, non-significant; ***, *P* < 0.001. | | | | | | | | | |

| Table S2. Amino acid profile (mg/ g) of *longissimus thoracis* muscle of female and male cattle, yak, camel and horse (n = 9). | | | | | | | | | |
| --- | --- | --- | --- | --- | --- | --- | --- | --- | --- |
| Indices | Sex | Species | | | |  | *P*-values | | |
|  |  | Cattle | Yak | Camel | Horse |  | Species | Sex | Species × Sex |
| Met | M | 0.51±0.02^b^ | 0.65±0.03^a^ | 0.49±0.03^b^ | 0.50±0.03^b^ |  | *** | NS | NS |
|  | F | 0.54±0.01^b^ | 0.66±0.02^a^ | 0.52±0.04^b^ | 0.51±0.01^b^ |  |  |  |  |
| Val | M | 0.91±0.02 ^b^ | 1.14±0.03 ^a^ | 0.88±0.03 ^b^ | 0.89±0.05 ^b^ |  | *** | NS | NS |
|  | F | 0.93±0.02 ^b^ | 1.13±0.01 ^a^ | 0.91±0.05 ^b^ | 0.92±0.00 ^b^ |  |  |  |  |
| Lys | M | 1.84±0.08 ^b^ | 2.11±0.05 ^a^ | 1.79±0.05 ^b^ | 1.79±0.09 ^b^ |  | *** | NS | NS |
|  | F | 1.87±0.02 ^b^ | 2.08±0.04 ^a^ | 1.84±0.13 ^b^ | 1.83±0.02 ^b^ |  |  |  |  |
| Ile | M | 0.89±0.03 ^b^ | 1.03±0.03 ^a^ | 0.86±0.02 ^b^ | 0.87±0.06 ^b^ |  | *** | NS | NS |
|  | F | 0.91±0.01 ^b^ | 1.04±0.03 ^a^ | 0.87±0.05 ^b^ | 0.90±0.01 ^b^ |  |  |  |  |
| Phe | M | 0.91±0.03 | 0.94±0.02 | 0.90±0.03 | 0.89±0.06 |  | NS | NS | NS |
|  | F | 0.90±0.02 | 0.90±0.03 | 0.95±0.06 | 0.93±0.01 |  |  |  |  |
| Leu | M | 1.55±0.05 ^b^ | 1.89±0.06 ^a^ | 1.53±0.04 ^b^ | 1.56±0.08 ^b^ |  | *** | NS | NS |
|  | F | 1.60±0.02 ^b^ | 1.87±0.02 ^a^ | 1.55±0.10 ^b^ | 1.60±0.01 ^b^ |  |  |  |  |
| Thr | M | 0.84±0.03 ^b^ | 1.02±0.04 ^a^ | 0.83±0.03 ^b^ | 0.83±0.05 ^b^ |  | *** | NS | NS |
|  | F | 0.87±0.01 ^b^ | 0.99±0.06 ^a^ | 0.85±0.06 ^b^ | 0.86±0.00 ^b^ |  |  |  |  |
| His | M | 0.68±0.01 ^c^ | 1.00±0.03 ^a^ | 0.68±0.04 ^c^ | 0.79±0.14 ^b^ |  | *** | NS | NS |
|  | F | 0.71±0.04^b^ | 0.96±0.02 ^a^ | 0.75±0.04 ^b^ | 0.88±0.07 ^a^ |  |  |  |  |
| Asp | M | 0.67±0.02 ^c^ | 2.00±0.06 ^a^ | 1.71±0.05 ^b^ | 0.69±0.03 ^c^ |  | *** | NS | NS |
|  | F | 0.70±0.02 ^c^ | 2.09±0.05 ^a^ | 1.76±0.11 ^b^ | 0.71±0.01 ^c^ |  |  |  |  |
| Ser | M | 3.19±0.14 ^a^ | 0.85±0.01 ^c^ | 0.66±0.03 ^d^ | 2.99±0.11 ^b^ |  | *** | NS | NS |
|  | F | 3.24±0.05 ^a^ | 0.90±0.05 ^c^ | 0.70±0.05 ^d^ | 3.03±0.05 ^b^ |  |  |  |  |
| Glu | M | 0.71±0.03 ^c^ | 3.48±0.04 ^a^ | 3.11±0.08 ^b^ | 0.68±0.01 ^c^ |  | *** | NS | NS |
|  | F | 0.68±0.03 ^c^ | 3.60±0.03 ^a^ | 3.14±0.22 ^b^ | 0.69±0.01 ^c^ |  |  |  |  |
| Pro | M | 0.82±0.03 ^a^ | 0.84±0.03 ^a^ | 0.69±0.03 ^b^ | 0.77±0.02 ^a^ |  | ** | NS | NS |
|  | F | 0.79±0.01 ^a^ | 0.85±0.02 ^a^ | 0.73±0.05 ^b^ | 0.82±0.03 ^a^ |  |  |  |  |
| Gly | M | 1.10±0.04 ^a^ | 0.90±0.03 ^b^ | 0.74±0.04 ^c^ | 1.07±0.04 ^a^ |  | *** | NS | NS |
|  | F | 1.11±0.02 ^a^ | 0.90±0.02 ^b^ | 0.78±0.04 ^c^ | 1.09±0.01 ^a^ |  |  |  |  |
| Ala | M | 1.75±0.07 ^a^ | 1.28±0.02 ^b^ | 1.05±0.04 ^c^ | 1.70±0.10 ^a^ |  | *** | NS | NS |
|  | F | 1.78±0.03 ^a^ | 1.31±0.02 ^b^ | 1.09±0.07 ^c^ | 1.74±0.01 ^a^ |  |  |  |  |
| Tyr | M | 0.67±0.03 ^bc^ | 0.85±0.02 ^a^ | 0.66±0.01 ^c^ | 0.73±0.04 ^b^ |  | *** | NS | NS |
|  | F | 0.72±0.01 ^bc^ | 0.83±0.02 ^a^ | 0.66±0.05 ^c^ | 0.75±0.01 ^b^ |  |  |  |  |
| Arg | M | 1.32±0.05 ^b^ | 1.42±0.03 ^a^ | 1.28±0.05 ^b^ | 1.25±0.06 ^b^ |  | ** | NS | NS |
|  | F | 1.33±0.03 ^b^ | 1.49±0.02 ^a^ | 1.33±0.10 ^b^ | 1.29±0.01 ^b^ |  |  |  |  |
| TAA | M | 18.37±0.64 ^b^ | 21.40±0.05 ^a^ | 17.85±0.58 ^b^ | 18.00±0.97 ^b^ |  | *** | NS | NS |
|  | F | 18.69±0.27 ^b^ | 21.60±0.11 ^a^ | 18.44±1.18 ^b^ | 18.55±0.06 ^b^ |  |  |  |  |
| EAA | M | 7.45±0.26 ^b^ | 8.78±0.09 ^a^ | 7.28±0.22 ^b^ | 7.34±0.42 ^b^ |  | *** | NS | NS |
|  | F | 7.62±0.11 ^b^ | 8.68±0.05 ^a^ | 7.50±0.49 ^b^ | 7.54±0.04 ^b^ |  |  |  |  |
| NEAA | M | 10.92±0.38 ^b^ | 12.61±0.13 ^a^ | 10.57±0.36 ^b^ | 10.67±0.55 ^b^ |  | *** | NS | NS |
|  | F | 11.07±0.16 ^b^ | 12.92±0.07 ^a^ | 10.94±0.70 ^b^ | 11.01±0.02 ^b^ |  |  |  |  |
| FAA | M | 5.56±0.21 ^c^ | 9.08±0.10 ^a^ | 7.88±0.25 ^b^ | 5.38±0.24 ^c^ |  | *** | NS | NS |
|  | F | 5.60±0.12 ^c^ | 9.38±0.07 ^a^ | 8.10±0.53 ^b^ | 5.52±0.03 ^c^ |  |  |  |  |
| EAA/TAA | M | 40.54±0.01 | 41.05±0.48 | 40.79±0.09 | 40.74±0.15 |  | NS | NS | NS |
|  | F | 40.78±0.04 | 40.17±0.11 | 40.66±0.05 | 40.65±0.08 |  |  |  |  |
| EAA/NEAA | M | 68.18±0.04 | 69.66±1.36 | 68.89±0.26 | 68.74±0.42 |  | NS | NS | NS |
|  | F | 68.85±0.10 | 67.16±0.31 | 68.51±0.15 | 68.50±0.23 |  |  |  |  |
| FAA/TAA | M | 30.28±0.23 ^c^ | 42.45±0.37 ^b^ | 44.13±0.03 ^a^ | 29.93±0.27 ^c^ |  | *** | NS | NS |
|  | F | 29.98±0.24 ^c^ | 43.42±0.27 ^b^ | 43.93±0.13 ^a^ | 29.78±0.12 ^c^ |  |  |  |  |
| The values of the above indicators were generated as individual traits in terms of species and sex. a-d Indicates the differences in the same row with significance at *P* < 0.05. TAA, total amino acids; EAA, essential amino acids; NEAA, non-essential amino acids; FAA, flavor amino acids, including Asp, Glu, Gly, Ala and Arg; M, male; F, female; NS, non-significant; **, *P* < 0.01; ***, *P* < 0.001. | | | | | | | | | |

| Table S3. Fatty acid profile (% of total FA) of *longissimus thoracis* muscle of female and male cattle, yak, camel and horse (n = 9). | | | | | | | | | |  |
| --- | --- | --- | --- | --- | --- | --- | --- | --- | --- | --- |
| Indices | Sex | Species | | | |  | *P*-values | | |  |
|  |  | Cattle | Yak | Camel | Horse |  | Species | Sex | Species × Sex |  |
| C14:0 | M | 8.00±0.35 ^a^ | 2.08±0.09 ^d^ | 3.20±0.25 ^c^ | 4.47±0.78 ^b^ |  | *** | NS | NS |  |
|  | F | 7.19±0.31 ^a^ | 2.07±0.09 ^d^ | 2.93±0.05 ^c^ | 4.51±0.30 ^b^ |  |  |  |  |  |
| C16:0 | M | 27.31±0.80 ^ab^ | 14.25±0.30 ^c^ | 26.81±0.10 ^b^ | 30.16±0.77 ^a^ |  | *** | NS | NS |  |
|  | F | 29.10±0.33 ^a^ | 14.85±0.38 ^c^ | 25.18±0.21 ^b^ | 28.85±0.32 ^a^ |  |  |  |  |  |
| C18:0 | M | 15.05±1.67 ^c^ | 21.56±0.28 ^b^ | 27.86±0.61 ^a^ | 3.40±0.14 ^d^ |  | *** | NS | NS |  |
|  | F | 14.56±0.35 ^c^ | 21.57±0.28 ^b^ | 27.03±1.93 ^a^ | 3.60±0.16 ^d^ |  |  |  |  |  |
| **SFAs** | | M | 52.90±1.60 ^a^ | 39.85±0.36 ^b^ | 55.96±0.93 ^a^ | 37.72±1.73 ^b^ |  | *** | NS | NS |
|  | | F | 50.38±1.11 ^a^ | 38.15±0.26 ^b^ | 50.68±5.86 ^a^ | 35.04±0.33 ^b^ |  |  |  |  |
| C14:1 | M | 0.11±0.03 ^c^ | 0.33±0.06 ^a^ | 0.16±0.01 ^b^ | 0.29±0.02 ^a^ |  | *** | NS | NS |  |
|  | F | 0.14±0.02 ^b^ | 0.33±0.05 ^a^ | 0.18±0.02 ^b^ | 0.30±0.04 ^a^ |  |  |  |  |  |
| C16:1 | M | 2.74±0.24 ^c^ | 5.03±0.16 ^b^ | 2.40±0.16 ^c^ | 6.47±0.06 ^a^ |  | *** | NS | NS |  |
|  | F | 3.32±0.26 ^c^ | 5.04±0.16 ^b^ | 3.19±0.21 ^c^ | 7.11±0.13 ^a^ |  |  |  |  |  |
| C17:1 | M | 0.31±0.03 ^b^ | 0.68±0.03 ^a^ | 0.32±0.02 ^b^ | 0.38±0.04 ^b^ |  | *** | NS | NS |  |
|  | F | 0.29±0.01 ^b^ | 0.66±0.03 ^a^ | 0.33±0.04 ^b^ | 0.32±0.04 ^b^ |  |  |  |  |  |
| C18:1n9c | M | 37.20±1.68 ^b^ | 45.38±0.68 ^a^ | 31.67±1.59 ^c^ | 34.92±0.87 ^b^ |  | *** | NS | NS |  |
|  | F | 38.21±1.02 ^b^ | 45.39±0.70 ^a^ | 34.01±1.18 ^c^ | 36.34±0.79 ^b^ |  |  |  |  |  |
| C20:1n9 | M | 0.25±0.05 ^b^ | 0.35±0.03 ^a^ | 0.13±0.02 ^d^ | 0.17±0.01 ^c^ |  | *** | NS | NS |  |
|  | F | 0.26±0.07 ^a^ | 0.34±0.03 ^a^ | 0.14±0.01 ^b^ | 0.15±0.03 ^b^ |  |  |  |  |  |
| C22:1n9 | M | 0.58±0.92 ^b^ | 0.50±0.09 ^b^ | 4.39±0.05 ^a^ | 5.58±1.54 ^a^ |  | *** | NS | NS |  |
|  | F | 0.71±0.50 ^c^ | 0.42±0.18 ^c^ | 4.79±0.08 ^b^ | 6.85±0.47 ^a^ |  |  |  |  |  |
| C24:1n9 | M | 2.02±0.01 ^b^ | 0.15±0.03 ^c^ | 3.01±0.49 ^a^ | 3.55±0.32 ^a^ |  | *** | NS | NS |  |
|  | F | 1.80±0.02 ^b^ | 0.15±0.04 ^c^ | 3.42±0.78 ^a^ | 4.02±0.45 ^a^ |  |  |  |  |  |
| **MUFAs** | | M | 41.12±1.60 ^c^ | 53.82±0.92 ^a^ | 34.64±1.66 ^d^ | 46.07±1.69 ^b^ |  | *** | NS | NS |
|  | | F | 43.88±1.31 ^b^ | 50.86±0.57 ^a^ | 41.03±6.07 ^b^ | 51.02±1.84 ^a^ |  |  |  |  |
| C18:2n6c | M | 2.07±0.19 ^b^ | 2.81±0.07 ^a^ | 2.53±0.73 ^ab^ | 3.03±0.10 ^a^ |  | *** | NS | NS |  |
|  | F | 1.34±0.07 ^b^ | 2.82±0.05 ^a^ | 1.35±0.46 ^b^ | 2.98±0.21 ^a^ |  |  |  |  |  |
| C18:3n6 | M | 0.50±0.03 ^b^ | 0.40±0.03 ^b^ | 0.96±0.40 ^b^ | 8.88±0.08 ^a^ |  | *** | NS | NS |  |
|  | F | 0.46±0.02 ^b^ | 0.50±0.03 ^b^ | 0.53±0.09 ^b^ | 5.81±0.42 ^a^ |  |  |  |  |  |
| C20:2 | M | 0.02±0.01 ^b^ | 0.10±0.05 ^a^ | ND | 0.05±0.01 ^b^ |  | * | NS | NS |  |
|  | F | 0.02±0.01 ^b^ | 0.11±0.04 ^a^ | ND | 0.04±0.01 ^b^ |  |  |  |  |  |
| C20:3 | M | 0.36±0.01 ^a^ | 0.34±0.03 ^a^ | 0.11±0.04 ^c^ | 0.19±0.01 ^b^ |  | ** | NS | NS |  |
|  | F | 0.32±0.13 ^ab^ | 0.38±0.02 ^a^ | 0.08±0.01 ^c^ | 0.17±0.01 ^b^ |  |  |  |  |  |
| C20:4n6 | M | 0.10±0.03 ^c^ | 1.20±0.05 ^a^ | 0.37±0.21 ^b^ | 0.18±0.03 ^b^ |  |  | NS | NS |  |
|  | F | 0.06±0.01 ^d^ | 1.21±0.05 ^a^ | 0.28±0.05 ^b^ | 0.16±0.05 ^c^ |  |  |  |  |  |
| C20:5n3 | M | ND | 0.24±0.05 ^a^ | 0.24±0.10 ^a^ | 0.04±0.01 ^b^ |  | ** | NS | NS |  |
|  | F | ND | 0.23±0.05 ^a^ | 0.12±0.04 ^a^ | 0.06±0.01 ^b^ |  |  |  |  |  |
| C22:6n3 | M | 0.62±0.11 ^a^ | 0.48±0.01 ^a^ | 0.07±0.02 ^b^ | 0.04±0.01 ^b^ |  | *** | NS | NS |  |
|  | F | 0.40±0.13 ^a^ | 0.49±0.01 ^a^ | 0.03±0.01 ^b^ | 0.04±0.00 ^b^ |  |  |  |  |  |
| **PUFAs** | M | 2.99±0.24 ^c^ | 5.41±0.38 ^b^ | 4.40±0.97 ^b^ | 9.71±1.92 ^a^ |  | *** | NS | NS |  |
|  | F | 3.14±0.25 ^c^ | 6.22±0.37 ^b^ | 2.59±0.65 ^c^ | 8.94±0.52 ^a^ |  |  |  |  |  |
| SFAs/UFAs | M | 1.28±0.08 ^b^ | 0.70±0.03 ^c^ | 1.57±0.06^a^ | 0.62±0.06 ^c^ |  | *** | NS | NS |  |
|  | F | 1.15±0.05 ^a^ | 0.64±0.02 ^b^ | 1.07±0.21 ^a^ | 0.67±0.04 ^b^ |  |  |  |  |  |
| PUFAs/SFAs | M | 0.05±0.00 ^c^ | 0.15±0.01 ^b^ | 0.07±0.02 ^c^ | 0.23±0.05 ^a^ |  | *** | NS | NS |  |
|  | F | 0.05±0.00 ^c^ | 0.15±0.01 ^b^ | 0.05±0.00 ^c^ | 0.27±0.01 ^a^ |  |  |  |  |  |
| The values of the above indicators were generated as individual traits in terms of species and sexes. a-d Indicates the differences in the same row with significance at *P* < 0.05. SFAs, total saturated fatty acids; MUFAs, total monounsaturated fatty acids; PUFAs, total polyunsaturated fatty acids; UFA, unsaturated fatty acids; M, male; F, female; ND, not detected; NS, non-significant; *, *P* < 0.05; **, *P* < 0.01; ***, *P* < 0.001. | | | | | | | | | |  |

| Table S4. The processing characteristics of *longissimus thoracis* muscle of female and male cattle, yak, camel and horse (n = 9). | | | | | | | | | |
| --- | --- | --- | --- | --- | --- | --- | --- | --- | --- |
| Indices | Sex | Species | | | |  | *P*-values | | |
|  |  | Cattle | Yak | Camel | Horse |  | Species | Sex | Species × Sex |
| pH | M | 5.86±0.02^A^ | 5.76±0.08^AB^ | 5.75±0.05^A^ | 5.71±0.01^B^ |  | *** | NS | NS |
|  | F | 5.83±0.03^A^ | 5.84±0.05^A^ | 5.82±0.03^A^ | 5.75±0.03^B^ |  |  |  |  |
| Cooking loss | M | 40.87±1.46^AB^ | 35.47±1.22^C^ | 39.08±0.84^B^ | 44.65±0.60^A^ |  | *** | NS | NS |
| (%) | F | 42.06±1.40^AB^ | 35.48±0.47^C^ | 39.07±0.36^B^ | 44.16±0.63^A^ |  |  |  |  |
| Shear force | M | 69.94±2.17^Bx^ | 78.64±3.41^Ax^ | 75.66±0.96^AB^x | 55.27±1.11^Cx^ |  | * | *** | NS |
| (N/cm^2^) | F | 55.89±1.17^y^ | 55.62±4.90^y^ | 49.91±2.24^y^ | 51.16±1.30^y^ |  |  |  |  |
| Marbling score | M | 3.20±0.48^By^ | 2.80±0.47^ABy^ | 4.85±0.50^Ay^ | 2.80±0.36A^By^ |  | *** | *** | * |
|  | F | 4.80±0.48^Bx^ | 6.80±0.48^ABx^ | 8.80±0.40^Ax^ | 6.80±0.50^ABx^ |  |  |  |  |
| *L^*^* | M | 35.15±0.44^A^ | 28.34±0.52^B^ | 33.80±0.58^Ay^ | 29.70±0.32^By^ |  | *** | * | NS |
|  | F | 33.39±0.51^B^ | 30.86±1.43^B^ | 35.57±0.17^Ax^ | 31.29±0.98^Bx^ |  |  |  |  |
| *a^*^* | M | 13.94±0.54^B^ | 15.63±0.82^A^ | 10.14±0.09^C^ | 12.66±0.11^Bx^ |  | *** | ** | NS |
|  | F | 13.16±1.01^B^ | 15.85±0.07^A^ | 12.67±0.16^B^ | 9.79±0.37^Cy^ |  |  |  |  |
| *b^*^* | M | 11.05±0.28^A^ | 10.62±0.41^A^ | 9.50±0.59^By^ | 11.05±0.28^A^ |  | *** | *** | *** |
|  | F | 10.58±0.36 | 10.62±0.57 | 11.70±0.48^x^ | 10.63±0.46 |  |  |  |  |
| Visual color | M | 3.88±0.13^B^ | 4.56±0.15^Ax^ | 3.75±0.28^Bx^ | 4.75±0.15^Ax^ |  | *** | *** | *** |
|  | F | 3.50±0.25^B^ | 4.02±0.10^Ay^ | 3.00±0.23^By^ | 4.28±0.13^Ay^ |  |  |  |  |
| The data of indicators of meat processing attributes were generated as individual traits in terms of species and sexes. A-C Different letters indicate significant differences (*P* < 0.05) among species while x-y between sexes. M, male; F, female; NS, non-significant; *, *P* < 0.05; **, *P* < 0.01; ***, *P* < 0.001. | | | | | | | | | |
